# Intravenous Delivery of HIV-Based Lentiviral Vectors Preferentially Transduces F4/80+ and Ly-6C+ Cells in Spleen, Important Target Cells in Autoimmune Arthritis

**DOI:** 10.1371/journal.pone.0055356

**Published:** 2013-02-04

**Authors:** Ben T. van den Brand, Eline A. Vermeij, Claire E. J. Waterborg, Onno J. Arntz, Michael Kracht, Miranda B. Bennink, Wim B. van den Berg, Fons A. J. van de Loo

**Affiliations:** 1 Rheumatology Research and Advanced Therapeutics, Department of Rheumatology Radboud University Nijmegen Medical Centre, Nijmegen, The Netherlands; 2 Rudolf-Buchheim-Institute of Pharmacology, Justus-Liebig-University Giessen, Giessen, Germany; Maisonneuve-Rosemont Hospital, Canada

## Abstract

Antigen presenting cells (APCs) play an important role in arthritis and APC specific gene therapeutic targeting will enable intracellular modulation of cell activity. Viral mediated overexpression is a potent approach to achieve adequate transgene expression levels and lentivirus (LV) is useful for sustained expression in target cells. Therefore, we studied the feasibility of lentiviral mediated targeting of APCs in experimental arthritis. Third generation VSV-G pseudotyped self-inactivating (SIN)-LV were injected intravenously and spleen cells were analyzed with flow cytometry for green fluorescent protein (GFP) transgene expression and cell surface markers. Collagen-induced arthritis (CIA) was induced by immunization with bovine collagen type II in complete Freund's adjuvant. Effect on inflammation was monitored macroscopically and T-cell subsets in spleen were analyzed by flow cytometry. Synovium from arthritic knee joints were analyzed for proinflammatory cytokine expression. Lentiviruses injected via the tail vein preferentially infected the spleen and transduction peaks at day 10. A dose escalating study showed that 8% of all spleen cells were targeted and further analysis showed that predominantly Ly6C+ and F4/80+ cells in spleen were targeted by the LV. To study the feasibility of blocking TAK1-dependent pathways by this approach, a catalytically inactive mutant of TAK1 (TAK1-K63W) was overexpressed during CIA. LV-TAK1-K63W significantly reduced incidence and arthritis severity macroscopically. Further histological analysis showed a significant decrease in bone erosion in LV-TAK1-K63W treated animals. Moreover, systemic Th17 levels were decreased by LV-TAK1-K63W treatment in addition to diminished IL-6 and KC production in inflamed synovium. In conclusion, systemically delivered LV efficiently targets monocytes and macrophages in spleen that are involved in autoimmune arthritis. Moreover, this study confirms efficacy of TAK1 targeting in arthritis. This approach may provide a valuable tool in targeting splenic APCs, to unravel their role in autoimmune arthritis and to identify and validate APC specific therapeutic targets.

## Introduction

Inflammatory diseases, such as rheumatoid arthritis, are characterized by infiltration of leukocytes into the inflamed tissue consisting of various immune cells from both the innate and adaptive immune system. Macrophages in arthritis have been extensively studied and play an important role in maintaining inflammation and joint destruction [1]; [2]. Moreover, a general approach of macrophage specific targeting with lipoplexes ameliorates experimental arthritis [3]; [4].

Specifically modulating macrophage activity during inflammatory conditions is a suitable approach to limit systemic side effects of treatment. In addition, gene therapy can be a powerful technique to achieve intracellular modulation of protein expression or signaling. Effective gene therapeutic treatment of collagen-induced arthritis has been achieved by ectopic overexpression of suppressor of cytokine signaling 3 (SOCS3) in splenic antigen presenting cells (APCs) [5]. In addition, knock down of TNF receptor I by overexpression of a short hairpin was also effective in ameliorating experimental arthritis [6]. Both studies used adenoviral vectors, ensuring a high, but transient transgene overexpression. Over recent years, HIV based lentiviral vectors have been optimized regarding safety and production and proved to be a valuable tool in order to get long term transgene expression.

Multiple studies have evaluated the feasibility of intravenous injection of lentivirus. These studies shown that the spleen is one of the organs predominantly targeted by lentivirus injected intravenously [7]. Further analysis on localization on GFP expression in the spleen showed large fluorescent clusters overlapping the marginal zone [8]. Cell surface markers revealed that mostly MHC-II positive cells were targeted by lentivirus [8]; [9], indicating preferential targeting of APCs by lentivirus.

Specific targeting of APCs by lentivirus makes it possible to study the role of these APCs in inflammatory conditions. In addition, specific interference with gene expression can possibly identify new therapeutic targets in APCs. To validate macrophage targeting a known potent therapeutic target was used, TGF-beta activated kinase 1 (TAK1). This MAP kinase is a key signaling protein as it is used by many pro-inflammatory signals. Toll-Like receptor and cytokine receptor activates intracellular signaling which converges to TAK1 and subsequently activate AP-1 and NF-κB. Therefore, TAK1 poses a compelling target to block pro-inflammatory signaling. Moreover, TAK1 has been shown to be important in autoimmune arthritis [3] and is a suitable target to validate macrophage specific targeting by lentiviral vectors.

In this study, we validated intravenous lentivirus injections into DBA1/J mice by determining expression kinetics, a dose escalating study, and analysis of targeted cells. This has shown that predominantly splenic F4/80+ and Ly-6C+ cells are targeted by this approach. To evaluate the gene therapeutic potential of LV-mediated targeting of splenic APCs, a TAK1 kinase negative mutant (TAK1-K63W) was over expressed during collagen-induced arthritis. TAK1-K63W decreased knee joint inflammation macroscopically and systemic Th17 levels in the spleen were reduced. In addition, bone erosion was diminished by TAK1-K63W and pro-inflammatory cytokine expression in synovial tissue was decreased. This study shows that intravenous delivery of lentiviral vectors enables evaluation of therapeutic targets in splenic monocyte/macrophage-like cells that are important cells in autoimmune diseases.

## Materials and Methods

### Ethics Statement

All in vivo studies complied with national legislation and were approved by local authorities (Animal Ethics Committee, Radboud University Nijmegen. Permit number: 2009-177) for the care and use of animals with related codes of practice.

### Mice

Male DBA/1 mice aged 10–12 weeks (Janvier, Elavage, France) were housed in top-filter cages and fed a standard diet with freely available food and water.

### Induction and monitoring of collagen-induced arthritis

Bovine type II collagen was dissolved in 0.05 M acetic acid to a concentration of 2 mg/ml and was emulsified in equal volumes of Freund's complete adjuvant (2 mg/ml of Mycobacterium tuberculosis strain H37Ra) (Difco, Detroit, MI) Mice were immunized intradermally at the base of the tail with 100 ul of emulsion (50 ug of bovine type II collagen). Subsequently, mice were given an intra-peritoneal booster injection of 100 ug of type II collagen dissolved in phosphate buffered saline (PBS) on day 21. Two independent observers monitored clinical signs of arthritis in paws and ankle joints, macroscopically. Cumulative scoring based on redness, swelling, and, in later stages, ankylosis was as follows: 0 = no changes; 0.25 = 1–2 toes red or swollen; 0.5 = 3–5 toes red or swollen; 0.5 =  swollen ankle; 0.5 = swollen footpad; 0.5 = severe swelling and ankylosis, with a maximal score of 2 per paw.

### Plasmids

For generation of recombinant lentiviral vectors we used of the third-generation self-inactivating transfer vector pRLL-cPPT-PGK-mcs-PRE-SIN (PGK-empty) containing the human phosphoglyceratekinase (PGK) promoter (kind gift from J. Seppen, AMC Liver Center, Amsterdam, The Netherlands). For cloning we used cloned *Pfu* DNA polymerase (Stratagene, La Jolla, CA) and T4 DNA Ligase (New England Biolabs, Ipswich, MA). All generated constructs were verified by sequencing. The cDNA sequences of a kinase-inactive mutant of human TAK1 (K63W) were PCR cloned from pEGFP-C1-TAK1-K63W into *NheI/NsiI* sites of PGK-SIN using the following primers: RV 5′-ATGCATTCATGAAGTGCCTTGTCAG-3′, FW 5′-GCTAGCGCCACCATGTCGACA GCCTCCGCCGCC-3′ (non-tagged, Kozak sequence for enhanced translation introduced).

### Lentiviral vector production

Packaging of VSV-G pseudotyped recombinant lentiviruses was performed by transient transfection of 293T cells. One day prior to transfection, 293T cells were seeded in a T75 flask at 1×10^5^ cells/cm^2^ in DMEM supplemented with 10% FCS, 1 mM pyruvate, 40 µg/ml gentamicin and 0.01 mM water-soluble cholesterol (Sigma). Cells were co-transfected with 19 µg transfer vector, 14 µg *gag/pol* packaging plasmid (pMDL-g/p-RRE), 4.7 µg *rev* expression plasmid (RSV-REV) and 6.7 µg VSV-G expression plasmid (pHIT-G) by calcium phosphate precipitation. Transfections were performed in 6 ml DMEM without antibiotics and cholesterol and cultured for 16 hours. Thereafter medium was replaced with fully supplemented DMEM and supernatant harvested after 24 and 48 hours. Cell debris was removed by centrifugation at 1500 rpm for 5 minutes at 4°C, followed by passage through a 0.45 µm pore polyvinylidene fluoride Durapore filter (Millipore, Bedford, MA, USA). For concentration by ultracentrifugation 28 ml supernatant was overlaid on 4 ml 20% sucrose solution and centrifuged at 25.000 rpm for four hours in a Surespin 630 rotor (Thermo Fisher Scientific, Waltham, MA). Pelleted viruses were resuspended in sterile PBS and stored at −80°C. Viral titers were determined by assaying p24*^gag^* values with a commercial enzyme-linked immunosorbent assay (ELISA) kit (Abbott Diagnostics, Hoofddorp, the Netherlands) and expressed as ng p24*^gag^*/µl.

### In vivo imaging

*In vivo* bioluminescent imaging was performed on an IVIS Lumina system (Caliper Life Sciences, Hopkinton, MA, USA), 10 minutes after intraperitoneal injection of 150 mg/kg D-Luciferine (Caliper Life Sciences) dissolved in PBS. Mice were anesthetized with isoflurane/oxygen, placed on their back into the light tight chamber and imaged for 4 minutes with a sensitive CCD camera. Images taken were quantified using the Living Image 3.0 software (Caliper Life Sciences). Luciferase activity is presented in photons emitted per second per square cm.

### RNA isolation and quantitative PCR analysis

Spleen, synovium, and liver samples were disrupted using the MagNaLyser (Roche). Total RNA was extracted from the tissue homogenates and from cells using TRI reagent (Sigma) according to manufacturer's protocol. Isolated RNA was treated with DNAse followed by reverse transcription of 1 µg RNA into cDNA using Moloney murine leukemia virus reverse transcriptase 0.5 µg/µl oligo(dT) primers, and 12.5 mM dNTPSs (Invitrogen). Quantitative real-time PCR was performed using the StepOnePlus sequence detection system (Applied biosystems, Foster City, CA) PCR was performed in a total reaction volume of 12.5 µl consisting of appropriate cDNA. Five µM of forward and reverse primer and the sYBR green PCR master mix (Applied biosystems). PCR protocol consisted of 2 min at 50°C and 10 min of 95°C, followed by 40 cycles of 15 sec at 95°C and 1 min at 60°C. Quantification of PCR signals was achieved by calculating the difference between the cycle threshold value (Ct) of the gene of interest with the Ct value of their reference gene glycerldehyde-3-phophate dehydrogenase (GAPDH) for each sample (delta Ct)

### Histology

Spleens were isolated and fixed in phosphate buffered 4% paraformaldehyde and embedded in paraffin wax. Whole knee joints were dissected and fixed in phosphate buffered 4% paraformaldehyde followed by decalcification with 5% formic acid, and embedded in paraffin wax. Serial tissue sections (7 µm) were stained with Safranin O (BDH chemicals, Poole, UK) and counterstained with fast green (BHD Chemicals) or with hematoxylin/eosin (Merck, Germany) and eosin (Merck, Germany) (H&E). Serial sections were scored for histopathological changes on a 0-3 scale, by 2 independent observers in a blinded manner. Joint inflammation was determined by the presence of synovial cell infiltrates and inflammatory cell exudates. Connective tissue destruction was determined by cartilage and bone erosion.

### FACS analysis

Spleens from mice were mashed, filtered and erythrocytes were removed by osmotic shock and directly stained for GFP expression and CD3 (1∶200 eBioscience), CD19 (1∶50 BD Pharmingen), F4/80 (1∶400, Biolegend), and Ly-6C (1∶800, Biolegend) cell surface markers. For T-cell subset analysis in arthritic mice CD3^+^ cells were isolated from spleen using the Pan T cell Isolation Kit (Miltenyi Biotec, Germany) according to manufacturer instructions. Purified CD3^+^ cells were stimulated in RPMI 1640 (Invitrogen) supplemented with 10% FCS penicillin/streptomycin and pyruvate with 50 ng/ml PMA, 1 ul/ml/10^∧6^ cells Brefeldin A (BD Pharmingen) and 1 ug/ml ionomycin. After 4 hours of stimulation cells were stained with anti-mouse CD4-APC (1∶200) (BD Pharmingen) or IgG2a-APC control (BD Pharmingen) for 30 minutes at 4°C. Subsequently, cells were stained intracellular with anti-IL17-FITC and anti-IFNγ-PE or with their controls respectively IgG1-PE and IgG1-FITC according to instructions by manufacturer (BD Pharmingen). Stained cells were analyzed using FACScalibur (Becton Dickinson) and analyzed with FlowJow software.

### Bone marrow derived dendritic cells

Femurs and tibias of DBA1/J mice were flushed and bone marrow was harvested. After preparing a single cell suspension cells were plated (250,000 c ells per cm^2^) with 833 ng p24GAG equivalent and 20 ng/ml GM-SCF. Medium was changed on day 4 and day 8.

### Statistics

Statistical differences were determined by one-way ANVA or student's T-test (two sided) and GraphPad 5.0 software. P values below 0.05 were considered significant.

## Results and Discussion

### Expression kinetics is dependent on route of administration

**Figure 1:**
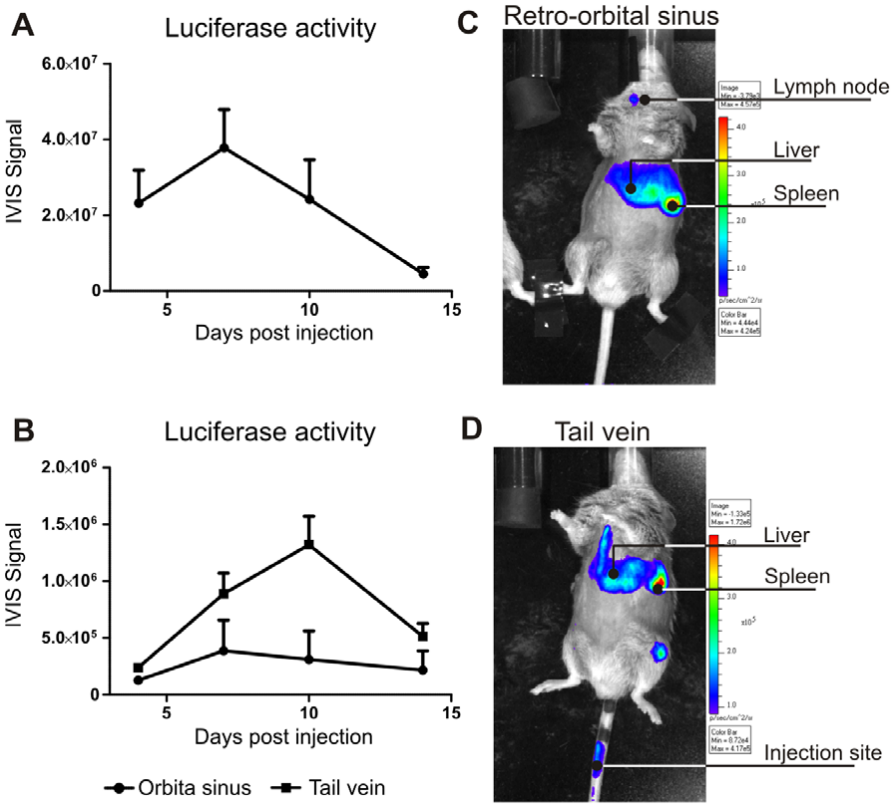
Transgene expression kinetics. Every animal received 10 µg of p24GAG equivalent of lentivirus encoding firefly luciferase intravenously. Luciferase activity was determined after injection of luciferine using biophotonic imaging with the IVIS Lumina. Region of interest was placed over abdomen of mice. Kinetics of transgene expression (A) and organ distribution of transduction (B) after lentivirus injection via the retro orbital sinus, n = 6. B). Comparison between retro orbital sinus and tail vein injection (n = 2 in both groups) for abdominal expression (C) and localization of transgene expression in tail vein injected animals (D).

Although previous studies have shown efficient transduction of spleen cells by intravenously delivered lentivirus, there are considerable differences between Balb/c and C57Bl/6 mice [10]. Therefore the kinetics of lentiviral mediated transgene expression needs to be determined in DBA1/J mice, an autoimmune prone strain used for the CIA model. We injected 10 µg p24GAG equivalent (approximately 5×10∧8 viral particles) of lentivirus encoding luciferase intravenously via the retro orbital sinus and luciferase activity was determined by in vivo imaging. Figure 1A shows that maximal luciferase activity was reached at day 7 after virus injection. Organ distribution of the luciferase activity is shown in Figure 1B and strikingly a substantial level of luciferase activity was found around the throat of the animals. These are possibly the submandibular lymph nodes draining the eye, but the precise localization was not identified. To avoid targeting this particular site and achieve more efficient delivery of LV to the spleen the retro orbital sinus injection site was compared with tail vein injections. Figure 1C shows that the expression kinetics of lentivirus injected via tail vein in spleen and liver are different from injection via retro orbital sinus. Maximal luciferase activity peaks at day 10 and expression levels of luciferase were elevated compared to retro orbital sinus injections. Tail vein injection also showed more luciferase activity in the liver and spleen area (Figure 1B and 1D). Unexpectedly, luciferase activity was also detected around the knee joint. This is most likely to originate from the bone marrow, as these cells are also transduced by lentivirus injected intravenously [7]; [10]; [11]. Additionally, these results were also observed with retro orbita plexus injections (data not shown). It appears that injection of lentivirus via the tail vein favors transgene expression levels and location and all subsequent experiments were performed with tail vein injections. Injection directly into a vein is potentially preferable over retro orbital sinus as the distribution of the lentivirus throughout the body is better than into a sinus, which has a slow blood flow.

### Dose escalating study of lentivirus

**Figure 2:**
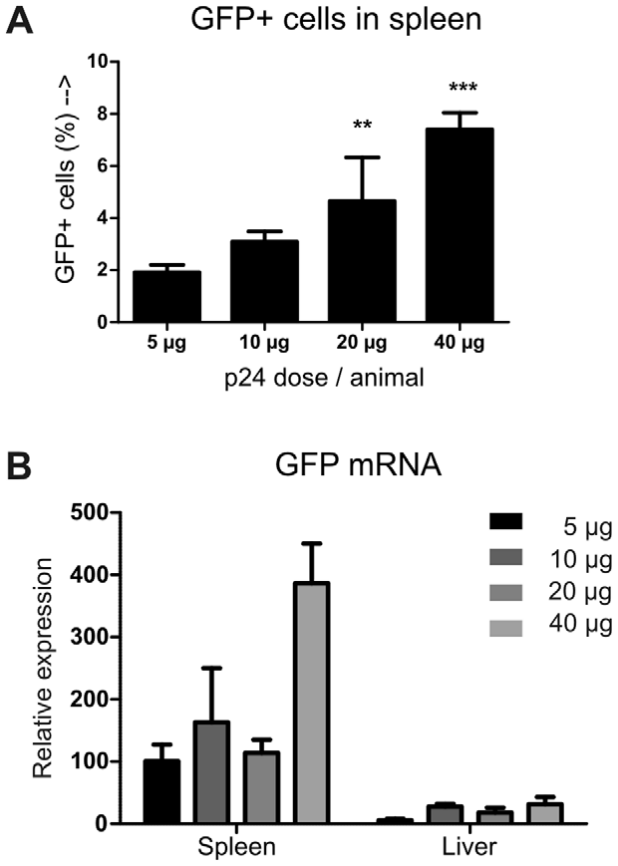
Dose finding of intravenous lentivirus injection. Increasing doses of lentivirus encoding GFP were injected via the tail vein and GFP expression was analyzed 10 days after injection. A) Total spleen cells were analyzed by flow cytometry for GFP expression. Statistics: one-way ANOVA with Bonferonni post test compared to PBS injected animals, n = 4 per group. ** p<0.01, *** p<0.001. B) GFP mRNA expression in liver and spleen samples. Relative expression  =  2^−ΔCt^. Data represented as mean±SEM.

After establishing the delivery route and transgene expression kinetics the optimal dose was determined by injecting increasing doses of lentivirus encoding GFP. Figure 2A shows that increasing doses of lentivirus also resulted in increasing percentage of GFP positive cells in spleen with 7.4% of total spleen cells were GFP positive when injected with 40 µg of p24GAG equivalent of lentivirus. Analysis of the GFP mRNA expression showed an increase in GFP mRNA with increasing dose of lentivirus, except at 10 µg lentivirus due to large variation. In addition, GFP mRNA expression showed a 12 fold increase in spleen compared to liver samples when administered 40 µg LV per animal (Figure 2B). Together with the luciferase activity measurements shown in Figure 1D we concluded that lentivirus efficiently transduces cells in the spleen. Although maximal expression of GFP was not reached we used 40 µg of p24GAG LV per animal in subsequent as a safe dose showing no side effects. GFP is known to elicit a specific immune response [12], but no increase in splenic cell number was observed in the dose escalating study (data not shown).

### Macrophages and monocytes are preferentially targeted by lentivirus

**Figure 3:**
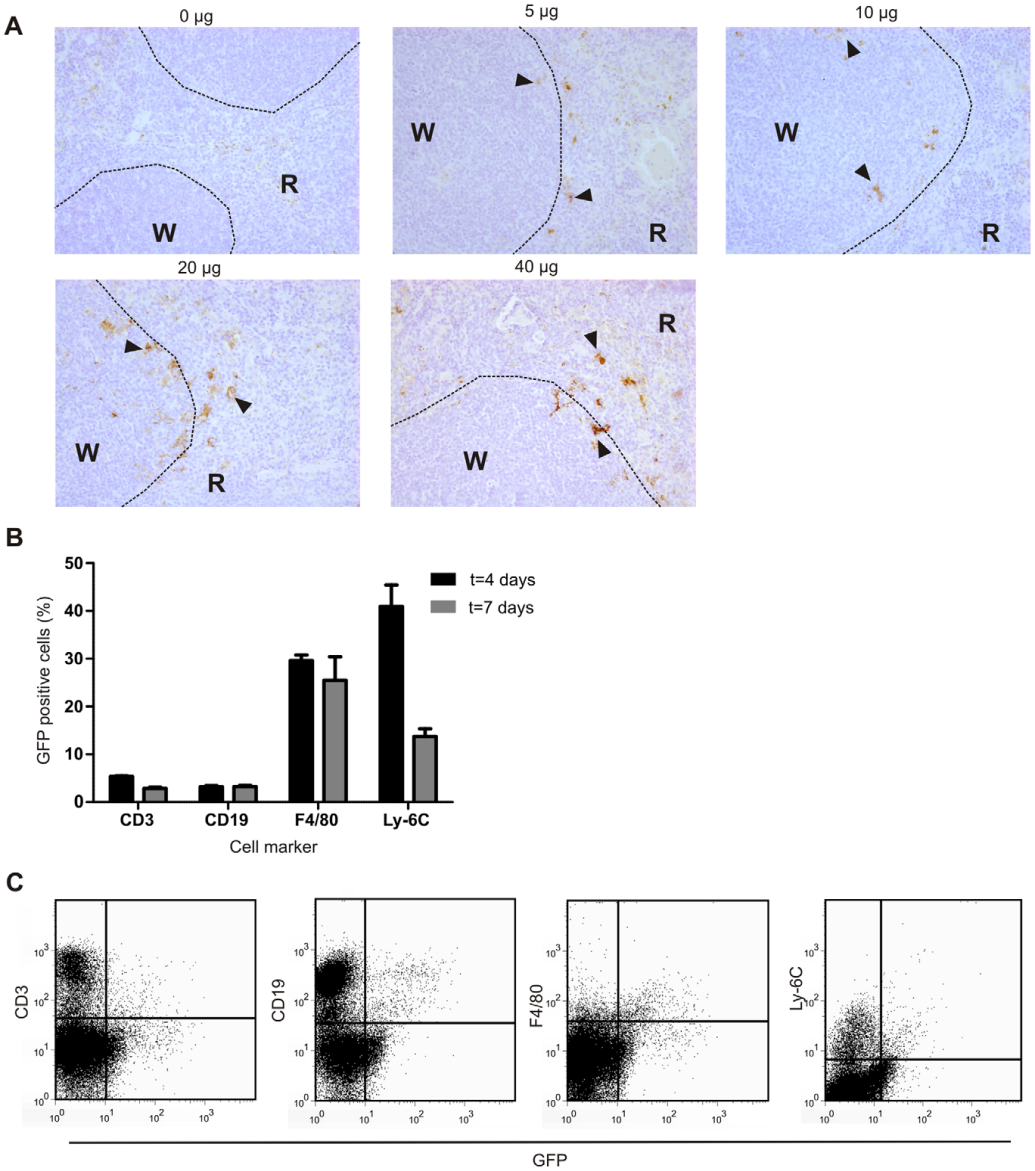
Localization splenic GFP expression and identification of transduced cell types by cell surface marker analysis. A) Increasing doses of lentivirus encoding GFP were injected via the tail vein and GFP expression was analyzed by immunohistochemistry ten days after virus injection. Representative histological images of experiment (magnification 200×), dotted line outlines white pulpa. W  =  white pulpa, R  =  red pulpa. Arrow heads indicate GFP positive cells. B) Flow cytometric analysis of GFP expressing cells 4 and 7 days after virus injection. Data represented as average (±SEM) GFP positive cells in gate indicated on x-axis (n = 4). C) Representative plots of cell marker and GFP FACS analysis.

Immunohistochemistry was done to determine the location of GFP expression in the spleen. Figure 3A shows that with increasing dose of lentivirus also more GFP protein is detected. GFP expression was detected around the white pulpa near the marginal zone, in line with other reports [8]; [13]. In a separate experiment it was determined which cells are targeted by lentivirus in the spleen, leukocytes were isolated from spleen and GFP expression was determined in combination with cell surface markers (Figure 3B). This confirmed that lentivirus only marginally targets T- and B-cells, since the IHC showed that there was no GFP expression in the white pulpa of the spleen. In contrast, it appears that lentivirus preferentially targets F4/80+ and Ly-6C+ cells as 30–40% of cell populations were GFP positive. The GFP expression in F4/80+ cells remains stable for up to ten days after virus injection (data not shown). This preferential targeting of the APC population has also been described by others [8]; [9]. The monocyte population shows a sharp drop off in GFP expression between day 4 and 7 after virus injection. This probably reflects migration or differentiation of Ly-6C^+^ monocytes, whereas the F4/80 expressing macrophages present in the red pulpa reside in the spleen [14]. The antigen presenting cell (APC) population in the spleen consists of many different subtypes of dendritic cells and macrophages. Here, we show a preferential targeting of the macrophage and monocyte population, but identification of the exact APC subpopulation remains to be determined.

### Lentivirus targets cells that play an important role during arthritis

**Figure 4:**
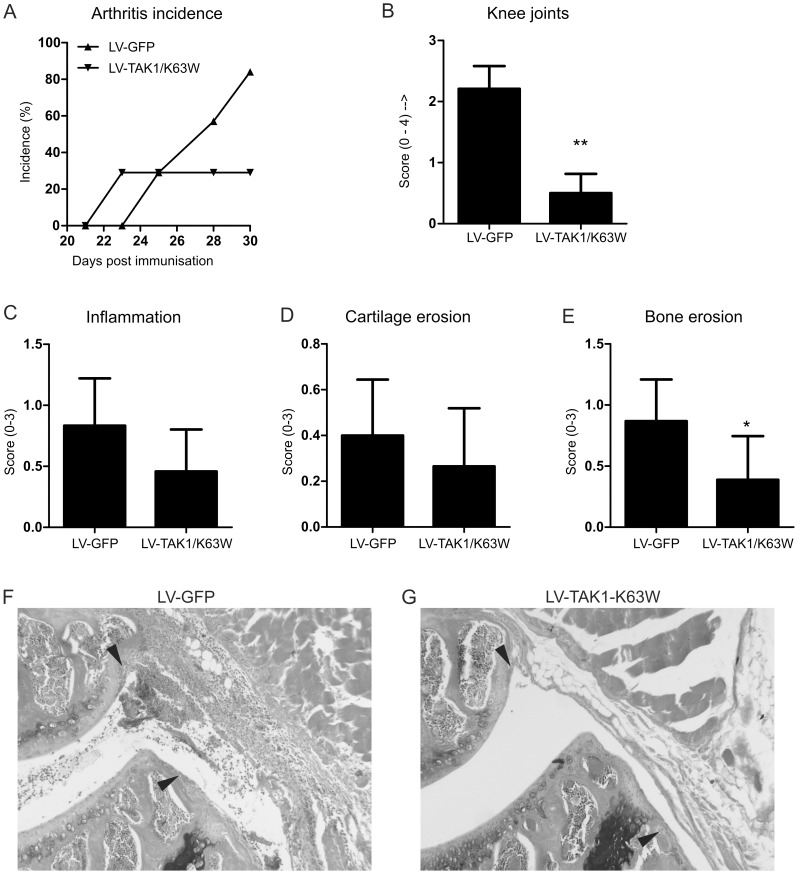
Effect of splenic TAK1 targeting on arthritis incidence and knee joint swelling. A) Arthritis incidence was monitored over time macroscopically. B) Swelling of knee joints macroscopically at time of sacrifice (day 30 of CIA). Data represented as mean±SEM. Statistics macroscopic scoring: Student's t-test, n = 7 per group. ** p<0.01. C–E) Knee joints were isolated and evaluated for pathohistological features. C) Inflammation scores determined on HE stained sections. D) Cartilage erosion scores determined on safranin-O stained sections. E) Bone erosion scores determined on safranin-O stained sections. F-G) Representative histological images for bone erosion of knee joints. Original magnification 100×. Bone erosion indicated with arrow head. P = patella, F = femur. Statistics histology: Student's t-test, n = 8 for GFP, n = 6 for TAK1-K63W. * p<0.05.

Next, we evaluated if the targeted cells also play a functional role in inflammatory diseases. Therefore mice immunized with bovine collagen type II were injected with lentivirus encoding a kinase negative mutant of TAK1 (TAK1-K63W) to decrease inflammatory signaling by Toll-Like Receptors and cytokines. This target was identified as an important signaling molecule in experimental arthritis and provides a suitable target to study the efficacy of intravenous injection of lentivirus [3]. Figure 4A shows that disease incidence is decreased in animals injected with lentivirus overexpressing the negative TAK1 mutant. In addition, macroscopic swelling of the knee joints was significantly decreased at time of sacrifice (Figure 4B). Further histological analysis (Figure 4C–E) showed only a trend towards decreased inflammation and cartilage erosion. Bone erosion, however, was significantly decreased in mice treated with TAK1-K63W. This is in line with the altered Th17 population, which plays an important role in osteoclast activity and bone erosion [15]. In addition, Ly-6C positive cells are precursors of osteoclasts [16] and inhibition of pro-inflammatory signaling on these cells possibly also inhibits osteoclast formation. A strong trend is found towards decreased levels of cell influx into the joint and Swirski and colleagues have described a pool of cells in the spleen that migrate towards the site of inflammation [17]. These cells were CD11b and Ly-6C positive and we have found Ly-6C+ cells expressing GFP after lentivirus administration (Figure 3B). Additionally, Ly6C-high expressing monocytes are attracted to the site of inflammation from the bone marrow in a kidney injury model [18]. Taken together, lentivirus injected intravenously could possibly target this pool of migrating monocytes in the spleen.

### Local inflammation decreased upon lentiviral targeting of macrophages and monocytes

**Figure 5:**
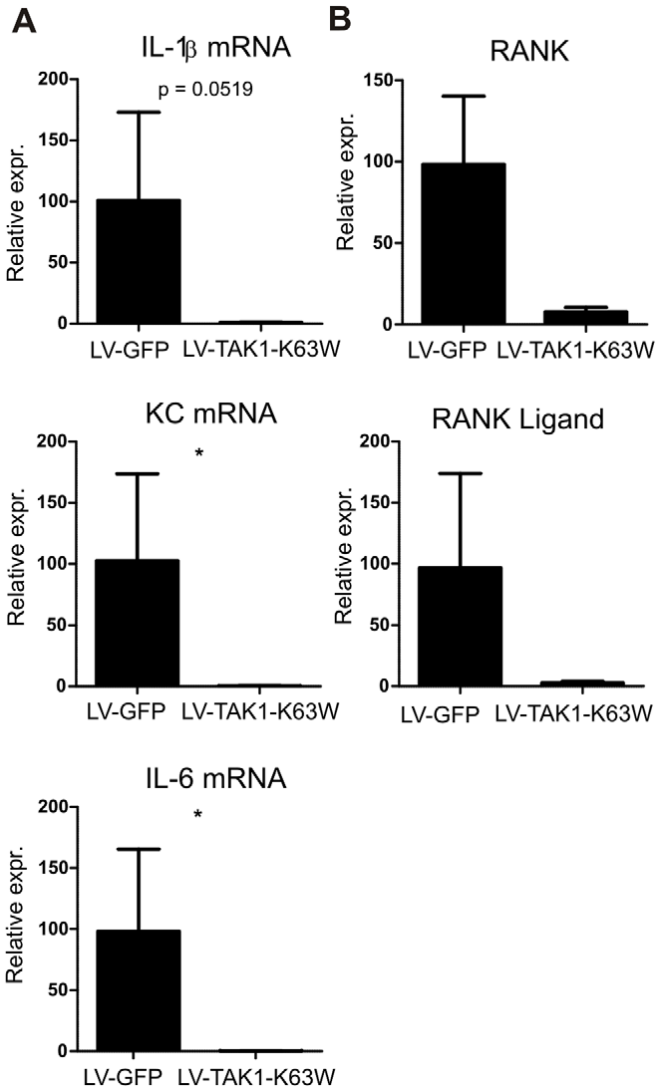
Proinflammatory cytokine expression in synovium of arthritic animals. Synovium was isolated from arthritic animals and disrupted. Total mRNA was isolated and analyzed for A) cytokines (IL-1β, IL-6, KC) and B) RANK, and RANKL mRNA expression. Data represented as mean±SEM. Relative expression  = 2^−ΔCt^. Statistics: Student's t-test, n = 6. * p<0.05.

**Figure 6:**
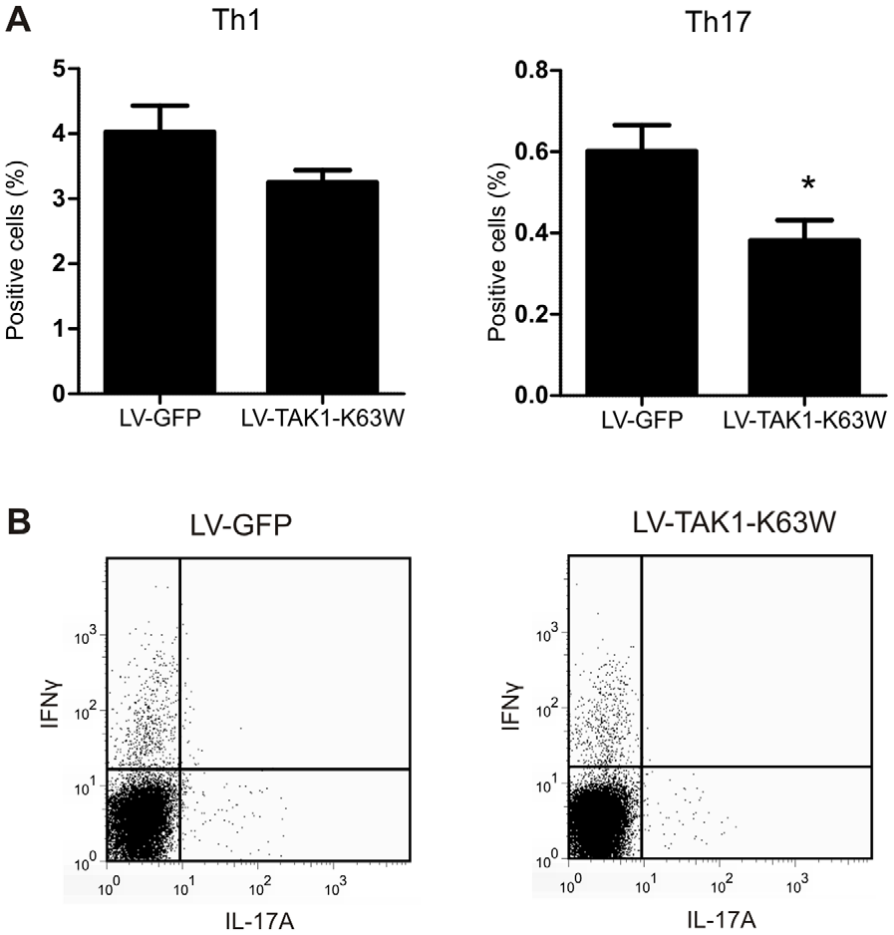
T-cell subsets in spleen during arthritis after TAK1 targeting in splenic APCs. A) Spleen cells were isolated and stimulated with PMA, ionomycin, and Brefeldin A for 4 hours and subsequently stained for CD4, IFNγ, and IL-17. Percent positive cells from CD4 gate is shown. B) Representative FACS analysis plots. IL-17 and IFNγ positive cells in CD4+ gate shown. Data represented as mean±SEM. Statistics: Student's t-test, two tailed, n = 7. * p<0.05.

From the inflamed joints synovium was extracted and mRNA analysis showed that proinflammatory cytokine mRNA expression was decreased when mice were treated with lentivirus encoding the TAK1 mutant (Figure 5). Although not significant, IL-1β production shows a trend towards diminished production in the inflamed synovium and might underlie the trend in decreased cartilage erosion. The decrease in IL-6 production also supports the diminished levels of Th17, as IL-6 is a crucial factor for Th17 development [19]. Moreover, RANK and RANK ligand, important for osteoclast formation and function are decreased in synovium of inflamed knee joints (Figure 6B) [20]. Together with altered systemic Th17 levels, this possibly causes the decrease in bone erosion. Additionally, production of the chemokine KC is significantly reduced and possibly explains the clear trend in reduced inflammation observed on histology. This shows that targeting the macrophages and monocytes in the spleen and liver can have distal effects on the inflamed tissue and shows the importance of this cell population in experimental arthritis.

### Lentivirus targets cells controlling the adaptive immunity

To study if the targeted cells are involved in controlling the adaptive immunity, T-cell subsets were determined in the spleen. At a systemic level, the TAK1 mutant had only marginally effects on Th1 development in the spleen. However, Th17 cell population was significantly decreased in the spleen by overexpression of the TAK1 mutant (Figure 6A). This suggests that lentivirus delivered intravenously also targets cells that are involved in controlling the adaptive immunity. This is in line with previous studies that showed transduction of MHC-II+ cells in spleen [9] and targeting of dendritic cell precursors and influence T-cell proliferation [21]. In addition, systemic lentivirus delivery has been used to induce tolerance in collagen-induced arthritis and showed clear potential in affecting T-cell function, with decreased proliferation [11].

### Bone marrow derived DCs express reduced levels of IL-12 and IL-23

**Figure 7:**
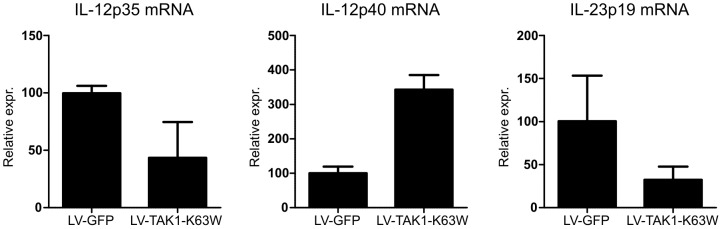
IL-12 and IL-23 mRNA expression decreased in bone marrow derived DCs. Bone marrow cells were harvested and transduced with either LV-GFP or LV-TAK1-K63W and subsequently differentiated into dendritic cells. After ten day differentiation period, mRNA was collected and analyzed for basal expression of IL-12p35, IL-12p40, and IL-23p19 using RT-PCR as described in Material and Methods. Data represented as mean±SEM, Relative expression  = 2^−ΔCt^, n = 2.

To evaluate how TAK1-K63W overexpression interferes with activation of the adaptive immunity we generated bone marrow derived DCs transduced with LV-GFP or LV-TAK1-K63W. These DCs were analyzed for IL-12p35, IL-12p40, and IL-23p19 mRNA expression and showed diminished levels of IL-12p35 and IL-23p19 and an increase in the IL-12p40 subunit (Figure 7). These decreased levels could cause the decrease in Th1 and Th17 development during the CIA. Additionally, the increase in IL-12p40 could enhance the inhibition on Th1 and Th17 development, since the IL-12p40 subunit can antagonize the function of IL-12 [22] and IL-23 [23]. These data show an important role for TAK1 in DCs in controlling the adaptive immunity.

TAK1 as therapeutic target in arthritis has been established by Courties and colleagues [3], in which TAK1 expression was knocked down using RNA interference and ameliorated collagen-induced arthritis. That study also showed altered T-cell subsets upon TAK1 targeting in APCs, but also showed a more efficacious therapy on arthritis severity. In the present study we overexpressed a negative mutant of TAK1 which is a competitive mutant and complete blockade of TAK1 cannot be achieved. In addition, lentivirus tropism is possibly more selective than lipoplexes. Nevertheless, we find matching results with TAK1 targeting in collagen-induced arthritis.

Interestingly, a novel role for TAK1 has been described recently [24]; [25]. Both studies have investigated the role of TAK1 in the production of pro-inflammatory cytokines and, remarkably, TAK1 negatively regulates cytokine production in myeloid cells. In these studies used lipopolysaccharide was used as stimulus, which is a very potent activator of neutrophils and macrophages. In addition, cytokine production was only determined over 24 hours. In contrast, collagen-induced arthritis involves a more complex process than a single stimulation and the clinical features of collagen-induced arthritis were studied for 12 days on our study.

In summary, this study shows an effective method to target a monocyte and macrophage population in spleen that is involved in inflammation and immunity. Blocking almost all pro-inflammatory signals, by overexpression of a TAK1 mutant, disrupts progression of experimental arthritis and impairs T-helper cell development. Injection of lentivirus intravenously provides a straightforward tool to study potential therapeutic targets in monocytes and macrophages during inflammatory conditions. Additionally, this study confirms the efficacy of TAK1 targeting in arthritis and emphasizes the role of this crucial MAP kinase in monocytes and macrophages during autoimmune arthritis.
